# Development of a Pretensioning Anchorage for Sand-Coated CFRP Tendons: Modeling and Validation

**DOI:** 10.3390/polym14245531

**Published:** 2022-12-17

**Authors:** Gian-Luca Züst, Valentin Ott, Giovanni Pietro Terrasi

**Affiliations:** Mechanical Systems Engineering Laboratory, Swiss Federal Laboratories for Materials Science and Technology, Überlandstrasse 129, CH-8600 Dübendorf, Switzerland

**Keywords:** clamping anchor, CFRP tendon, FE analysis, distributed fiber optic sensing

## Abstract

This paper presents a finite element (FE) analysis of an anchor for prestressing of sand-coated carbon-fiber-reinforced polymer (CFRP) tendons during the manufacturing of precast elements. This anchorage is temporary and removed after 2 to 7 days, when the pretensioning is released and the concrete is finally compressed. The investigated anchor consists of a conical metal barrel and three polymer wedges. The main tendon material properties are measured, compared with theoretical values and define the basis for the FE model. The latter considers both steps, pressing-in of the wedges and the subsequent loading of the tendon (diameter 7.5 mm). The relatively soft contact between polymer wedge and sand-coating is characterized experimentally and implemented with a pressure-overclosure condition. For the validation of the FE model, the strain distribution in the tendon is measured using fiber optical sensing. The therefore crucial process of fiber integration is described, and a novel approach is presented to uncover the optical fiber. The strain distribution of a sample with two anchors loaded in tension up to 80 kN is presented. The stress concentration at the front of the anchorage is highlighted. In addition, the finite element model is compared with the experiment, showing a good agreement of the strain distribution. A failure criterion according to Puck is finally implemented, which allows to assess potential fiber or interfiber failure.

## 1. Introduction

CFRPs have many advantages over steel. These are, for example, small density (1/5 of steel), high strength and therefore superior strength-to-weight ratio, excellent fatigue resistance, high corrosion resistance (even under sustained load), low thermal conductivity, high X-ray permeability, bad conduction of electricity and non-magnetizability. They have found more and more applications in prestressed reinforcements of civil engineering structures, where they induce a compressive stress on the concrete. Thus, the serviceability (stiffness and durability) of a concrete element subjected to tension, torsion or bending is enhanced. The higher the reinforcement is prestressed, the more prestress remains after the concrete has shrunk and crept after the curing process. In civil engineering, high-strength steel ($R_{p0.2}>1700$ MPa) reinforcements are often used in prestressed concrete structures. However, steel has a decisive disadvantage when it comes to lightweight construction. Steel needs a top concrete cover (of typically 50–65 mm depending on the exposure class and design standard considered [1]) to protect it from corrosion damage. Since steel is sensitive to environmental influences, protection of the steel surface is essential. This protective layer is called passivation. The pH value of the enclosing cement stone is essential for the passivation process. The pore liquid of the concrete has a pH value of about 13.0 to 13.8 [2]. The alkaline environment creates an oxide layer of iron atoms. This shell is only a few atomic layers thick (2–20 nm) but is an ideal protective layer for reinforcing steel against corrosion. However, as soon as the alkalinity decreases, the protective layer of iron atoms dissolves. Additionally, this can happen due to the carbonation of concrete under the influence of CO${}_{2}$ from the air. Therefore, the pH value of the pore liquid drops. From a pH value of around 11, the steel loses its passivity and begins to corrode under the influence of oxygen and H${}_{2}$O. The corrosion leads to an increase in volume of the steel surface layer and, therefore, also to tensile stresses in the surrounding concrete. This can lead to cracking or spalling of the concrete cover. A sufficiently thick concrete cover has been shown to protect steel reinforcement from external influences. This is a common practice in standards [1]. This additional concrete cover primarily fulfils a protective function and is, therefore, a non-structural component. This additional material and weight are accepted today. If this top layer thickness could be reduced, new applications would become possible. On the one hand, the material requirement would decrease, leading to economical and ecological benefits. On the other hand, the weight would be reduced, which simplifies the transport of precast elements. CFRP has the advantage over steel that it does not corrode, not even when subjected to sustained tensile stresses (CFRP does not suffer from stress corrosion). Therefore, if CFRP tendons replace the steel reinforcement, it is possible to reduce the concrete cover to the structurally required minimum and finally the weight of the prestressed concrete element.

Pretensioning of CFRP tendons require anchors, which are basically the critical element in the application of the promising material. CFRP are more attractive the higher they can be prestressed. However, local stress concentrations can lead to premature failures of this highly anisotropic material. Various methods for anchorage of FRP tendons were studied in the past [3,4]. Resin casting anchors and wedge anchors are two promising anchorage methods. However, they are only suitable for specific applications.

In casting anchors, a filled epoxy resin is poured into the barrel, where the CFRP tendon is positioned [5]. This anchor system so far reaches the best performance and finds applications in CFRP hangers for bridges [6]. The whole casting and curing procedure is laborious and time-consuming, and thus not economical for the manufacturing of precast elements. Therefore, wedge anchors, which were the state of the art for prestressing steel, were also introduced for fiber-reinforced polymers. They consist generally of a conical metal barrel and several wedges made of different materials [7].

In this paper, a wedge anchor is studied that clamps the CFRP tendon with three polymer wedges inserted in one steel barrel. Traditional pretensioning steels have ribs that create a form fitting connection with the concrete. CFRP tendons often make use of an applied sand coating, which provides bond to the concrete. The weak point when prestressing is always the anchorage itself. The anchoring system creates stress concentrations on the highly anisotropic tendon that cause failure at loads significantly lower than the tensile strength of the unidirectional CFRP material. Failure modes are often abrupt tensile failures with perpendicular fracture surface or pull-out failure by peeling of the sand layer or of the near-surface carbon fiber layer. On the other hand, shrinkage and creep of the concrete after prestress release reduce the effective precompression of the concrete. The highest possible prestress of typically 70% of the ultimate tensile CFRP tendon strength is sought-after [8,9].

Past research on the design of clamping anchors to grip and introduce sustained tensile loads in anisotropic FRP tendons was limited to finite element modeling validated with conventional strain measurements (resistive strain gauges mostly) [10,11]. Nellen et al. consider conventional fiber optic sensors based on Bragg gratings to locally measure strains [12]. No research is known where the longitudinal strain distribution is being measured in the clamped FRP area to validate the build-up of tension in the wedge anchor. This paper addresses this shortcoming with the use of distributed fiber optic sensing by integrating an optical fiber in the center of the CFRP tendon. In the paper by Terrasi et al. from 2011 [13], a numerical FEM of an anchorage system for sanded CFRP tendons of a diameter of 5.4 mm is presented. Other publications deal primarily with non-sanded tendons. They mainly examine the geometry of the anchors. In the paper by Heydarinouri et al., the influence of a differential angle between the wedge and the barrel is examined [14]. It was found that this differential angle can reduce the stress concentration on the CFRP tendon. This concept is well known today as it was introduced in FRP tendon anchorages first by Prof. Shrive of Queens University in Canada 25 years ago [15]. This paper presents a finite element model for assessing a wedge anchor to prestress a sand-coated d = 7.5 mm CFRP tendon and the validation of the internal strain profile with a distributed fiber optical sensor. The developed FE analysis reproduces the continuously measured strain with good accuracy and is capable to predict the strain peak at the loaded end of the anchor. This combined and innovative numerical-DFOS experimental approach will allow further optimization of the anchorage in the future by variation of its geometric as well as material parameters.

## 2. CFRP Material Properties

Comprehensive material characterization was performed for the tendon material consisting of a unidirectional carbon-fiber-reinforced epoxy cylindrical laminate of 7.5 mm diameter. This allowed improved modeling as well as quantitative assessment of the results with enhanced failure models. The tendon material parameters are listed in Table 1. Furthermore, the fiber volume content, the Young’s modulus in fiber direction and the tensile strength were experimentally determined. In addition, the compressive strength and the Young’s modulus perpendicular to the fibers were measured.

### 2.1. Fiber Volume Content

The fiber volume content $\psi_{f}$ was measured by sulphuric acid digestion according to DIN EN 2564, procedure B [16]. The measured value is close to the specified $\psi_{f}$ of 60% (see Table 2).

### 2.2. Young’s Modulus $E_{11}$

Based on the measured fiber volume content, the Young’s modulus can be calculated by the rule of mixture:

(1) $$E_{11}=\psi_{f}\cdot E_{f}+\left(1-\psi_{f}\right)\cdot E_{m}=0.6094\cdot 245\;\mathrm{GPa}+\left(1-0.6094\right)\cdot 2.85\;\mathrm{GPa}=150.6\;\mathrm{GPa}.$$

**Figure 1:**
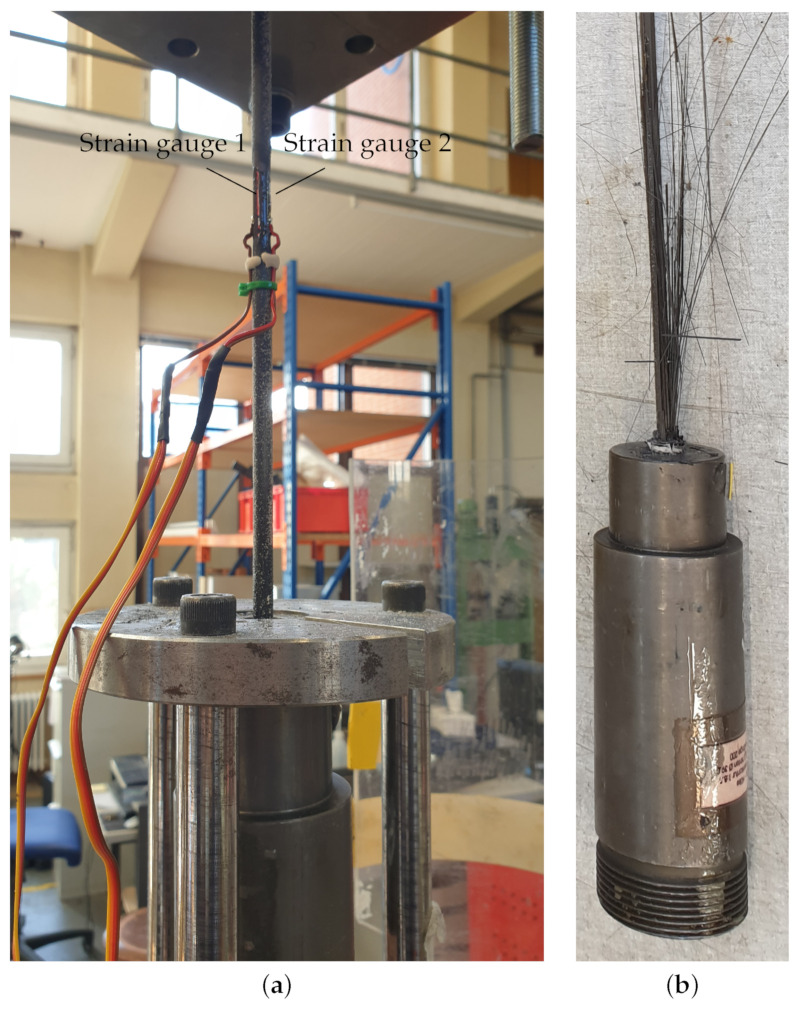
Tensile testing with resin cast anchor. (**a**) Sample with two strain gauges in the free length; (**b**) failed tendon showing partial fiber failure.

The Young’s modulus in fiber direction $E_{11}$ was measured according to the D7205/D7205M standard [17]. Two strain gauges of type HBM LY46-6/350 (Hottinger Baldwin Messtechnik, Darmstadt, Germany) were applied at the specimen mid-length, and the specimens were loaded in displacement-controlled mode with a rate of 2 mm/min (see Figure 1a). By the use of two strain gauges, potential bending strains were eliminated. The results in Table 3 reveal a relatively high variation due to the small measurement gauge length of 6mm on an anisotropic material. However, the average is close to the theoretical value calculated in Equation (1).

### 2.3. Tensile Strength with Resin-Cast Anchorage

The tensile strength was measured with conical barrels of 200 mm length with large interior diameter 39 mm and cone angle 6.87${}^{\circ }$. The cone was filled with the resin system of Huntsman LY 5052/Aradur® 5052 (Huntsman, Basel CH) and post-cured for 4 h at a temperature of 100 ${}^{\circ }$C. In theory, the theoretical strength can be calculated by the following formula:

(2) $$\sigma_{11_{ult}}\approx \psi_{f}\cdot \sigma_{f_{ult}}=0.6094\cdot 5000\;\mathrm{MPa}=3047\;\mathrm{MPa}.$$

This theoretical value will never be reached. Material supplier account for fiber fracture, misalignment and pores arising during pultrusion with a efficiency of 90% [18]). For the consideration of the tensile strength in fiber direction in Section 5, $R_{\left|\right|}^{+}$ = $\sigma_{11_{ult}}$·0.9 = 2742 MPa is therefore assumed.

Apparently, the measured strength values in Table 4 are lower than the calculated one of 2742 MPa. This can be explained by studying the failure pattern, where only partial fiber failure is present (see Figure 1b). The tested material has a relatively low fiber volume content and a highly ductile matrix, wherefore complete fiber failure is hard to achieve. The reduced load capacity can also be explained by the failure criteria described in Section 5. However, 93% of $R_{\left|\right|}^{+}$ is reached, which is considered to be an excellent result for a unidirectional CFRP tendon.

### 2.4. Compressive Strength in Transverse Direction

**Figure 2:**
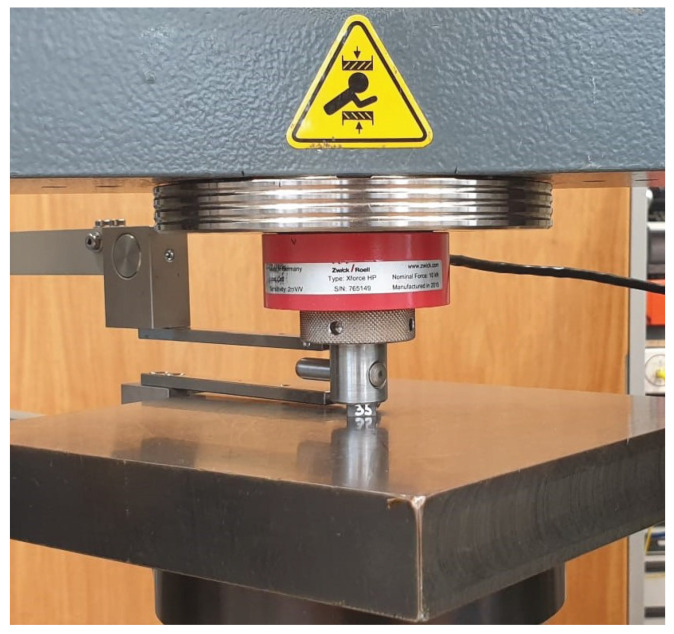
Experimental setup for compression test.

**Figure 3:**
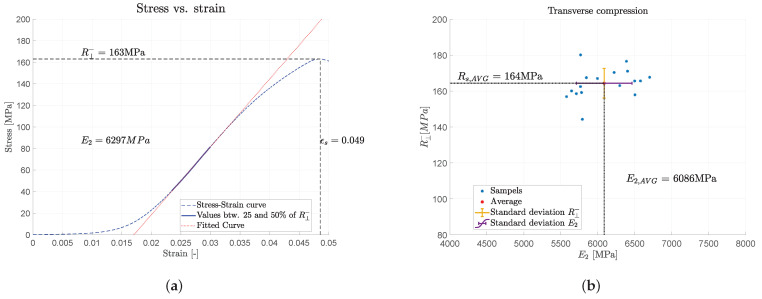
Transverse compression. (**a**) Typical stress–strain curve of one sample; (**b**) Scatter plot of 16 samples $E_{2}$ vs. $R_{⊥}^{-}$.

Compressive tests were performed on a universal testing machine Z010 (Zwick, Ulm, Germany) in order to determine the compressive strength and the Young’s modulus in transverse direction of the tendon. Therefore, samples with size 5 mm × 5 mm × 10 mm were cut from the tendon material. Deformations were measured with an extensometer between the plate and the upper socket of the test machine. The samples were loaded with a preload of 10 N and a speed of 0.1 mm/min (see Figure 2). A typical stress–strain curve of one sample is shown in Figure 3a. The Young’s modulus was determined between 25% and 50% of the ultimate stress. Test results of all samples with Young’s modulus $E_{2}$ versus the compressive strength in transverse direction $R_{⊥}^{-}$ are shown in Figure 3b. The average compressive strength is $R_{⊥}^{-}$=164 MPa, while the measured Young’s modulus is $E_{2}=E_{3}=6086\;\mathrm{MPa}$. This value is thought to be too small. Moreover, the stress–strain curve has a pronounced non-linear behavior. This can be explained by the specimen manufacturing tolerances of ±0.03 mm, where the two faces are never perfectly parallel.

The experimentally determined $E_{2}=E_{3}$ was compared with the formula according to Schneider et al. [19]:

(3) $$E_{2}=E_{3}=E_{m}^{\circ }\frac{\left(1+\psi_{f}^{3}\right)}{{\left(1-\psi_{f}\right)}^{0.75}+6\psi_{f}\frac{E_{m}^{\circ }}{E_{f}}}=7407\;\mathrm{MPa},$$

with:

(4) $$E_{m}^{\circ }=\frac{E_{m}}{1-v_{m}^{2}}=3274\;\mathrm{MPa}.$$

The values of the different variables were already introduced above. The Young’s modulus according to this formula seems to be more suitable than the measured one.

## 3. FE Model

**Figure 4:**
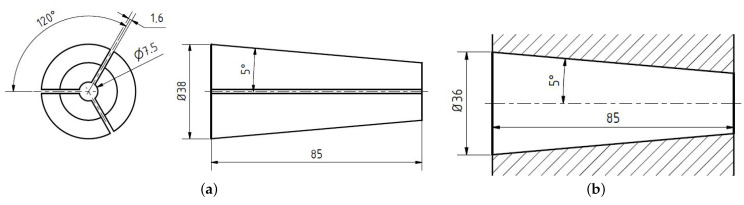
Sketches of the modeled wedges and the barrel. (**a**) Geometry of the wedge; (**b**) inner contour of the barrel.

**Figure 5:**
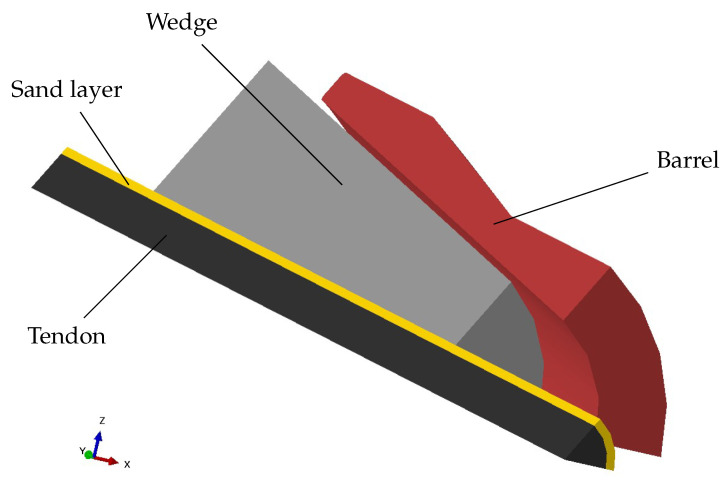
Reduced model simulated in Abaqus.

The FE analysis was performed with Abaqus/CAE 2021 (Dassault, Toulouse, France). The simulated anchor consists of a conical metal barrel (see Figure 4b) that anchors the sanded CFRP tendon with three polymer wedges (see Figure 4a). The wedges and the barrel have a cone half-angle of 5°. Thus, no differential angle was considered (as opposite to [13,14,15]). Both the wedge as well as the barrel have a length of 85 mm. The CFRP tendon has a diameter of 7.5 mm and is provided with a quartz sand layer thickness of 0.75 mm (grain size 0.1–0.6 mm). Symmetry with respect to the wedge symmetry plane was exploited in order to reduce the numerical effort (see Figure 5).

### 3.1. Modeling and Assembly

All material parameters are summarized in Table 5. The metal barrel consists of commercially available isotropic steel. The wedges are made of PA6GF30, a 30-weight-% glass fiber (GF)-reinforced Polyamide (PA) 6 (Ems Chemie, Domat-Ems, Switzerland, CH). The compression Young’s modulus and the Poisson’s ratio are specified in the data sheet [20]. Part of the tendon material constants were already derived in Section 2. The remaining tendon properties were obtained using the formulas of Chamis [21].

It is challenging to model the sand layer correctly in the FEM. In theory, one would have to model the individual grains of sand and their interaction with the resin and the polymer wedges. In the authors’ opinion, this is not appropriate as it is computationally intensive, and the result would be difficult to be verified. Therefore, it was decided to create an orthotropic layer that reflects the material properties of sand. The characteristic of the sand is only a supporting function in the radial direction as well as the shear transfer in axial direction from the tendon to the wedge. In order to decouple the individual sand grains from the tendon surface, $E_{1}$, $E_{2}$ and $G_{12}$ were set to 1 MPa. If these values were higher, they would add stiffness to the tendon, which is not appropriate. With an assumed serial connection of sand and epoxy, an estimated volume fraction of 40% sand, a Young’s modulus of sand of 72.5 GPa [22] and Young’s modulus of the matrix of 3 GPa, the radial Young’s modulus $E_{3}$ of the sand layer is 4866 MPa. The shear stiffness $G_{13}$, which is mainly responsible for the shear transfer, and $G_{23}$ = $G_{12}$, were set to 1000 MPa. A value of zero is assumed for the Poisson’s ratio in all directions due to the discrete nature of the sand grains.

### 3.2. Boundary Conditions

**Figure 6:**
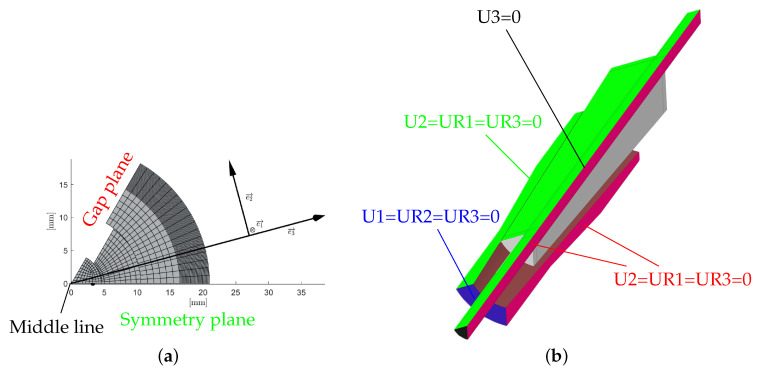
Definition of the boundary conditions. (**a**) Definition of planes and coordinate system; (**b**) visualization of boundary conditions.

A cylindrical coordinate system is suggested by the symmetry of the one-sixth model. Index 1 corresponds to the axial direction, index 2 to the tangential and index 3 to the radial direction. The definition of the coordinate system is shown in Figure 6a. The boundary conditions must fulfil the symmetry resulting from the reduced modeling. This results in four boundary conditions (see Figure 6b). The middle axis is fixed in radial direction (U3=0). The two cutting planes on the symmetry side and on the gap side must be fixed in tangential direction and are not allowed to rotate about the axes 1 and 3, which results in the condition U2=UR1=UR3=0. The barrel is not allowed to move in the axial direction and not to rotate about the in-plane axes 2 and 3. This results in the condition U1=UR2=UR3=0.

### 3.3. Interactions

There are three areas where the different parts interact which each other: barrel with wedge, wedge with sand layer and sand layer with the CFRP tendon. The latter interaction was solved with a tie, which represents the adhesive bonding of the sand on the tendon. The normal behavior between barrel and wedge is thought to be a “hard contact” and the tangential behavior a penalty with a friction coefficient of μ=0.2. The contact between the sand layer and the wedge is more sophisticated. Upon radial pressure, the relatively soft wedge penetrates the sand coating. This is necessary for the form fit connection between the wedge and the tendon. A sufficiently large friction coefficient μ takes this into account. Here, a friction coefficient μ=10 was selected. For the normal behavior, Terrasi et al. [13] considered a pressure-overclosure condition. This was repeated here but in a slightly different configuration, as described in the next paragraph. Table 6 summarizes all interactions.

#### 3.3.1. Procedure for Determining the Pressure–Overclosure Relationship

**Figure 7:**
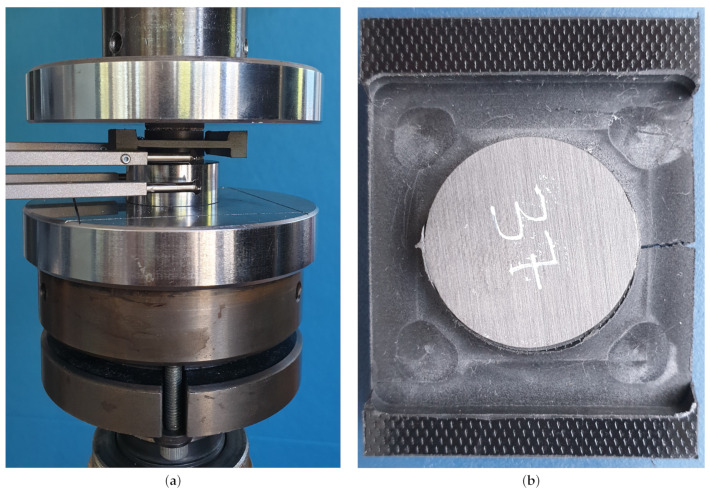

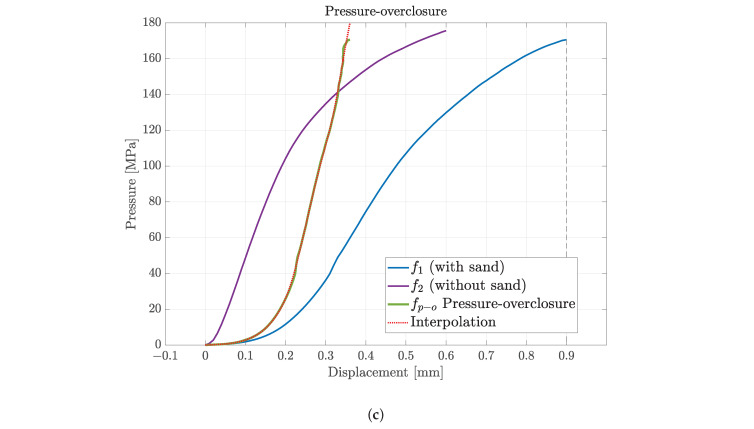
Experimental determination of the pressure-overclosure condition. (**a**) Test set for the compression test; (**b**) PA6GF30 plate under the sand-coated CFRP lamella disk: the plate failed at high compressive stresses; (**c**) pressure-overclosure condition for the configuration shown in (**a**).

A separate test setup was developed to measure the penetration of two sand-coated pultruded CFRP lamellae with uncoated thickness of 1.14 mm into an extruded polymer plate made of PA6GF30 with a thickness of 10.7 mm. The CFRP disks were sand-coated with quartz sand of size 0.1–0.6 mm. The force–displacement curve was measured with a universal testing machine Z1474 (Zwick, Ulm, Germany) and a load cell of ±100 kN. Reference measurements with non-coated CFRP disks were additionally performed in order to determine a pure pressure–overclosure relationship. Figure 7a shows the test setup and Figure 7b the specimens after the test. The curve determined was then smoothed with Matlab R2020b (MathWorks, Natick, MA, USA) and read into Abaqus via a tabular input. Figure 7c shows the measured curves and the interpolation of the pressure-overclosure condition.

#### 3.3.2. Derivation of This Assumption

The stack of the different material can be interpreted as a serial connection of springs with different stiffness. By making use of the superposition principle, the pressure overclosure can be derived. The total deformation is the sum of the individual deformed elements, which are loaded by the same force. The total deformation $∆t_{1}$ of the whole assembly with sand-coated CFRP disks as a function of the stress σ is given by $f_{1}\left(\sigma \right)$:

(5) $$∆t_{1}=f_{1}\left(\sigma \right)=2f{\left(\sigma \right)}_{disk}+2f{\left(\sigma \right)}_{Sand}+2f{\left(\sigma \right)}_{p-o}+f{\left(\sigma \right)}_{Plate}$$

In case of the reference configuration without sand, the sand term as well as the pressure-overclosure term disappear:

(6) $$∆t_{2}=f_{2}\left(\sigma \right)=2f{\left(\sigma \right)}_{disk}+f{\left(\sigma \right)}_{Plate}$$

The difference between these two values is the deformation due to the additional sand layer and the pressure overclosure caused by the sand penetrating the polymer plate:

(7) $$∆t=∆t_{1}-∆t_{2}=f_{1}\left(\sigma \right)-f_{2}\left(\sigma \right)=2f{\left(\sigma \right)}_{Sand}+2f{\left(\sigma \right)}_{p-o}.$$

The pressure–overclosure relation is derived by:

(8) $$f{\left(\sigma \right)}_{p-o}=\frac{1}{2}∆t-f{\left(\sigma \right)}_{Sand}$$

where

(9) $$f{\left(\sigma \right)}_{Sand}=\frac{\sigma }{E_{3}}\cdot t_{sand}$$

with the out-of-plane stiffness of the sand layer $E_{3}=4866$ MPa (see Table 5) and the thickness of the sand layer $t_{sand}$ = 0.75 mm. This term is neglected, as its contribution is very small compared with the pressure-overclosure term.

This test was analyzed using Matlab, and the results are illustrated in Figure 7c.

#### 3.3.3. Validation of the Pressure-Overclosure Condition

**Figure 8:**
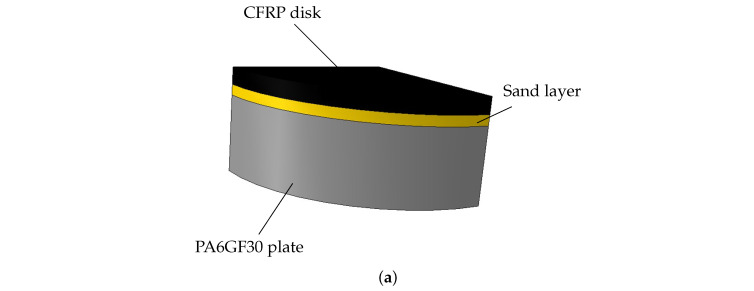

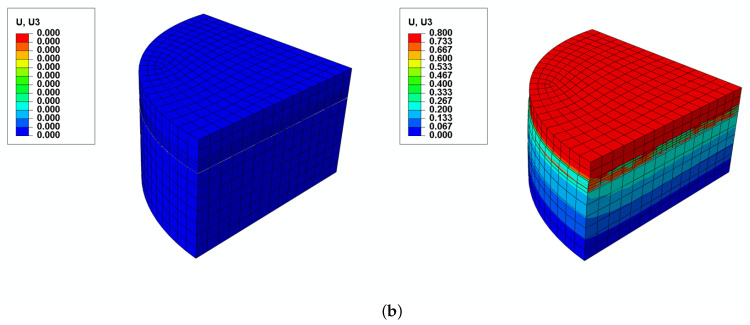
Model for validating the pressure-overclosure condition. (**a**) Quarter model with additional axial symmetry plane; (**b**) FE model; on the left: unloaded condition, the CFRP disk lies on the PA lamella. On the rigth: the CFRP disk is pressed into the PA lamella, and the penetration is well recognizable.

**Figure 9:**
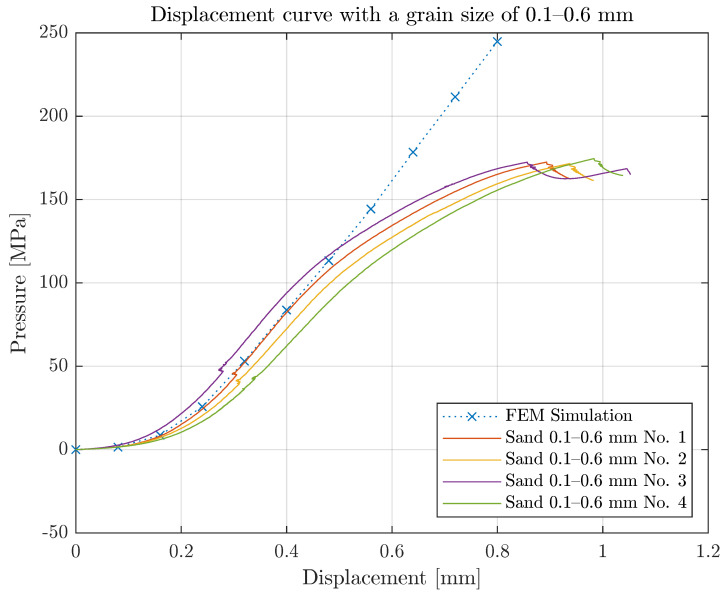
Comparison of modeled and measured pressure overclosure between CFRP lamella with sand at 0.1–0.6 mm and PA6GF30.

**Figure 10:**
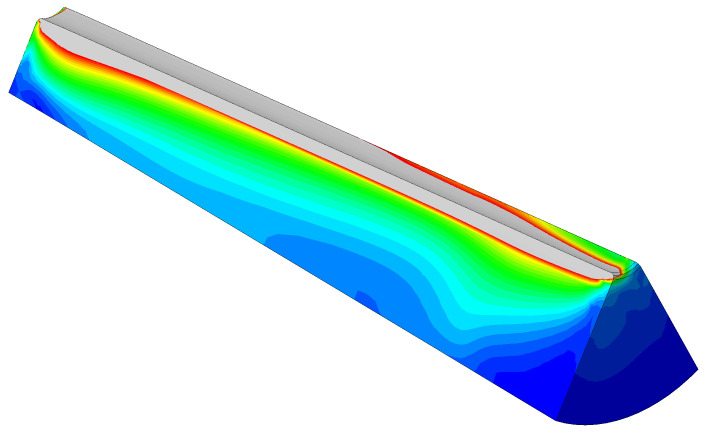
Stress according to the von Mises criterion, σ≥60 MPa colored gray.

This situation was also modeled in Abaqus in order to verify the pressure-overclosure condition. First, an FE model of the entire test setup was simulated, and the results were cross-compared with the measurement results. The definition and the model itself is shown in Figure 8 and the results in Figure 9. The model follows the measured values up to approximately 80 MPa. Above 80 MPa, the simulation result differs from the experiment. This is due to the significant non-linear material behavior of PA6GF30 above 80 MPa [20]. However, numerical simulations of the anchor described showed that the wedge is loaded locally with stresses up to 120 MPa. Despite this, significant creep is already observed at stresses above 60 MPa (see Figure 10), leading to viscoplastic deformations, which were neglected in the presented FE analysis. Characterization of creep and modeling was not in the scope of this research.

### 3.4. Mesh and Steps

The average mesh size was chosen to be 0.8 mm. The mesh of the tendon was refined to 0.4mm at the front and the back of the wedge. Stress concentrations are expected at these locations, wherefore the corners of the wedge were rounded and the mesh refined. Only elements of type C3D8 were used. The analysis consists of three steps. In the first step, the wedge is positioned with a small displacement in order to bring the different parts in contact and to avoid convergence problems in the beginning of the simulation. In the next step, the wedge is set with an axial load of 50 kN. This step is also necessary to initiate the form-fitting connection between the sand layer and the relatively soft wedge. In the loading step, the axial load on the wedge is removed and the tendon is pulled with a tensile stress up to the desired force.

## 4. Continuous Longitudinal Strain Measurement of the CFRP Tendon by Integrated DFOS

**Figure 11:**
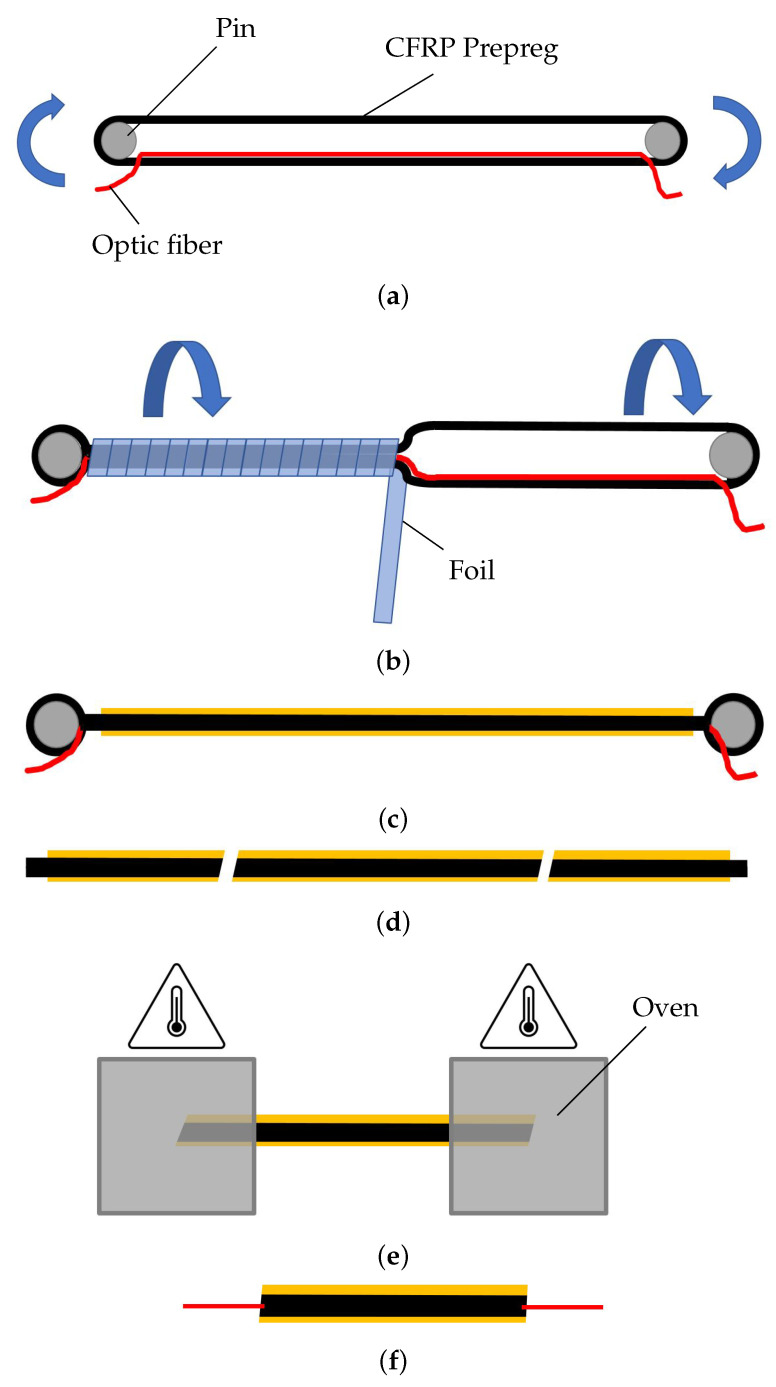
Procedure for manufacturing a CFRP tendon with an integrated optical fiber. (**a**) CFRP prepreg is wrapped around two pins, an optical fiber is placed in the middle of the loop; (**b**) the CFRP loop is rotated around its axis while being wrapped in foil; (**c**) the foil is removed after curing and the sand layer is applied; (**d**) the CFRP tendon is sawed into shorter pieces; (**e**) the matrix is decomposed in an oven by heating to 400${}^{\circ }$; (**f**) the exposed carbon fibers can be removed, and the optical fiber can be separated. The connection for the measuring device can then be spliced onto the end of the optical fiber.

**Figure 12:**
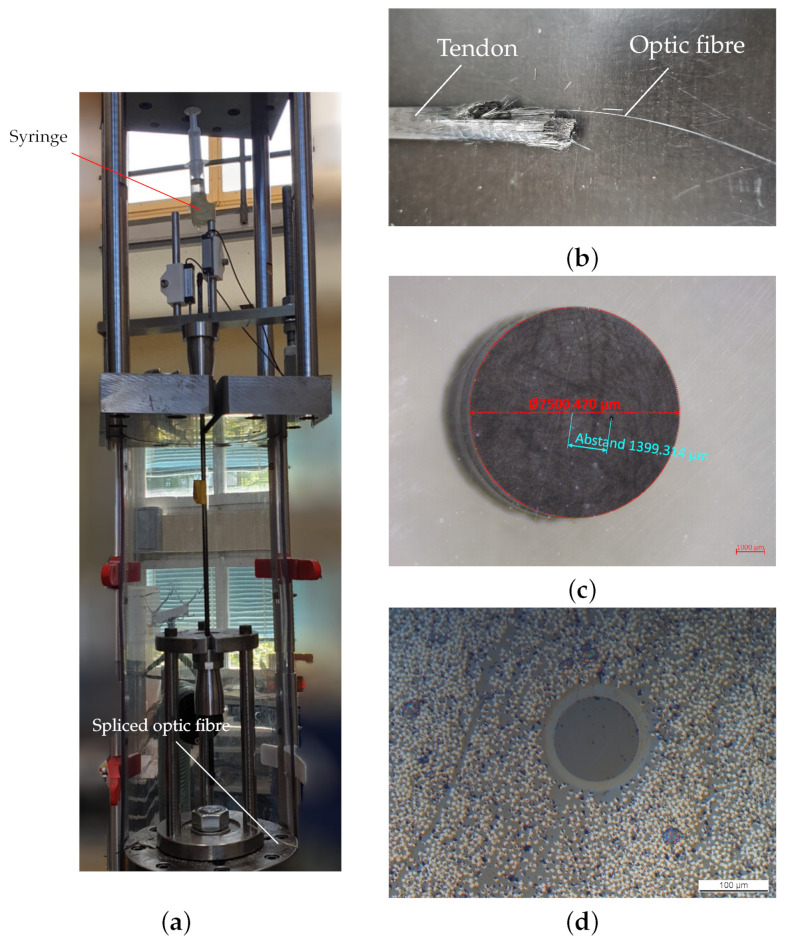
Tensile testing of anchored sample. (**a**) Setup for the fiber optic measurement; (**b**) prepared end of a CFRP tendon; (**c**) eccentricity of 1.4 mm of the optical fiber; (**d**) optical fiber with core and coating embedded in CFRP.

The integration of fiber Bragg sensors in CFRP tendons is state of the art. However, Nellen et al. [12] performed it in a pultrusion process, which is thought to be laborious for small sample lengths and relatively inflexible compared with the process presented here, where tendons with integrated optical fiber of the type Fibercore SM1250B3(9.8/125) [23] were manufactured (see Figure 11). The tendons were manufactured using a tape-laying process, where a unidirectional carbon fiber epoxy prepreg is wound around pins, and an optical fiber is placed between the upper and lower prepreg stack (see Figure 11a). Then, the lay-up is wrapped with polyester foil in order to consolidate the prepreg and to form a round cross-section (see Figure 11b). After the curing phase, the foil is removed, and the sand layer is applied as illustrated in Figure 11c. The tendon is cut into smaller pieces (Figure 11d). To extract and couple the optical fiber, the ends of the tendon are heated in an oven up to 400 °C, leading to decomposition of the epoxy matrix (Figure 11e). After that, the optical fiber has to be found in the bunch of dry carbon fibers (see Figure 11f). The result of this procedure is shown in Figure 12b.

Tensile tests were performed on a Instron 1343 universal test machine (Instron, High Wycombe UK) with a load cell of ± 250 kN (see Figure 12a).

**Figure 13:**
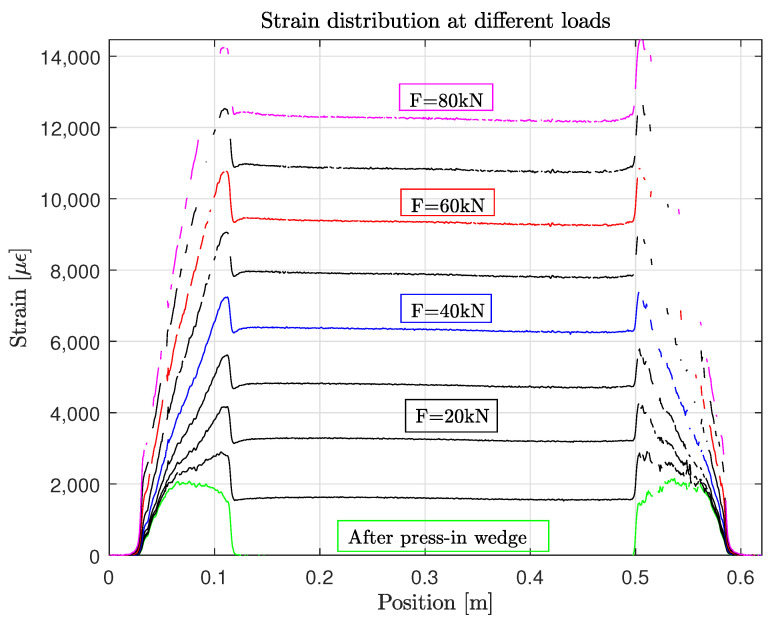
Strain distribution of the tendon at discrete load steps of 10 kN.

**Figure 14:**
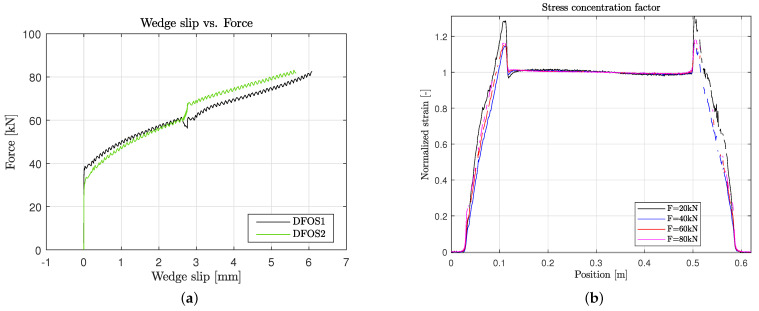
Wedge slip and stress concentration factor. (**a**) Wedge slip starting between 30 and 40 kN; (**b**) stress concentration at different loads.

The strain distribution along one test sample with a total length of 0.6 m and wedge anchors at both ends is shown in Figure 13. In general, the strain distribution curves have relatively good quality, which is due to the integration of the DFOS into the tendon. Correlation losses are not significant. In addition, the strain in the free length is constant. Moreover, the left anchor and the right one show similar patterns. The different curves correspond to different load levels. The zero measurement was taken before pressing in the wedge into the barrel. The strain after setting the wedge is in the range of 2000 μm. This stretching is caused by the radial pressure, which acts on the tendon. The anchorage length increases with increasing load. Above 40 kN, the strain gradient is almost constant. This also correlates with the measured wedge slip of two samples in Figure 14a, where the wedge slip starts between 30 kN and 40 kN. At higher loads, not only the strain is increased but also the stress concentration, due to the additional radial pressure caused by the wedge slip. By normalizing the strain distribution with the strain in the free length, a stress concentration factor can be estimated (see Figure 14b). At a force of 20 kN, this factor is the highest, which is still an effect of the previous setting of the wedge with a horizontal pressure of 50 kN. Interestingly, the stress concentration is constant for 40 kN, 60 kN and 80 kN and shows a value of approximately 17%. The clamped tendon failed in tension with a failure strain of 1.45% at the stress concentration location. This corresponds to a local stress of 2163 MPa, which is still much lower than the assumed tensile strength of $R_{\left|\right|}^{+}$ = $\sigma_{11_{ult}}$·0.9 = 2742 MPa. It is assumed that other damage mechanisms at the surface of the tendon are leading to failure, which is the result of the interaction of the wedge with the tendon and the sand coating (see Section 5).

**Figure 15:**
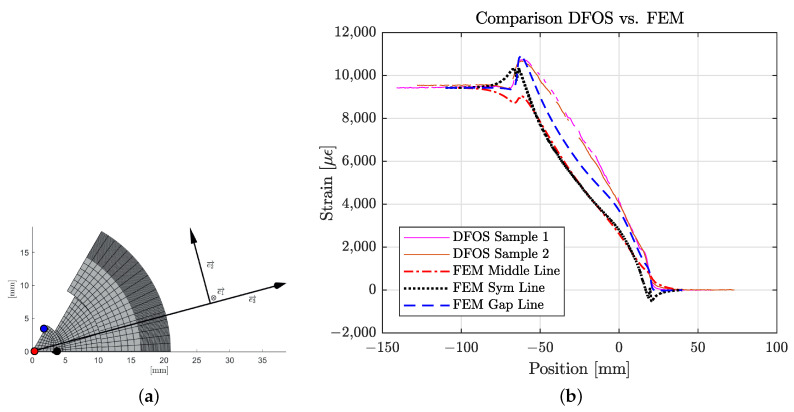
DFOS measurement vs. Finite Element Analysis. (**a**) Definition of analyzed points: (Middle line), (Gap line) and (Symmetry line); (**b**) strain distribution in the anchorage at σ=1400 MPa.

In the next step, the measured strain distribution inside the tendon was compared with the results of the FE analysis (see Figure 15b). Two measurements were taken (marked with “DFOS”). The scatter between the two measurements is small. The simulation was analyzed at three selected lines, which are illustrated in Figure 15a. The middle line runs precisely in the middle of the tendon and is, therefore, the axis of rotation. The symmetry line is located on the symmetry plane of the wedge on the second outermost node layer of the mesh of the CFRP tendon. The definition of the gap line is similar to the symmetry line but on the gap side.

The results show a good agreement with the measured ones. Quantitative deviations can be justified by some limitations of the model. Apparently, the model underestimates the stress concentration due to radial pressure of the wedge. It was also noticed that the wedge slip in the FE analysis was reduced compared with the experiment. This is thought to be due to the assumed linear elastic behavior of the wedge material. Non-linearities are relevant with these high preloads, as the wedges are highly deformed after tensile test. Moreover, the friction coefficient is hard to estimate and will be pressure-dependent. Wear of wedge material in the barrel testifies from hardly accountable processes in this contact. In addition, significant viscoplastic material behavior is observed.

One aspect is also the position of the optical fiber, which is not perfectly aligned in the middle. Microscopic inspection showed deviations up to 2 mm (see Figure 12c); at the same time, the diameter of the tendon is mainly affected by the sand coating and can vary ± 0.2 mm.

## 5. Failure Criteria Evaluation for the Tendon

**Figure 16:**
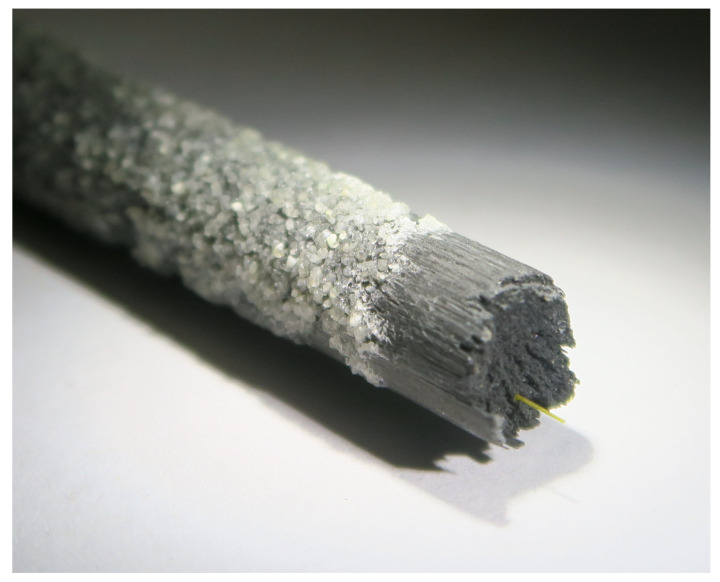
Quartz-sand-coated CFRP tendon showing the failure pattern with the integrated optical fiber.

The anchorage performance is assessed by the tensile strength. In order to optimize the design of the anchorage, an appropriate failure criterion is necessary. First, the tensile strength of three samples with one anchorage per side was tested. According to experience, the tensile strength is reduced compared with the result of the resin cast anchor in Section 2.3. With the wedge anchorage developed in this research, an average tensile stress of 1856 MPa was measured (see Table 7). This is 67.7% of the assumed tensile strength in the fiber direction of $R_{\left|\right|}^{+}=2742$ MPa. The typical failure pattern is shown in Figure 16.

This discrepancy needs to be explained by the help of a failure criterion. For a detailed analysis of the fracture behavior of the CFRP tendon, the failure criterion according to Puck was chosen. This criterion gives good insights into the possible failure mechanisms and was successfully applied for unidirectional CFRP tensile elements [24]. The puck criterion is more comprehensive compared with Tsai Wu [25] and Hashin [26], which are often implemented in commercial FE programs. In addition to matrix or fiber failure, this criterion also provides information about the interfiber failure mode. Furthermore, it predicts the orientation of the fracture plane.

In Puck’s failure criterion, fiber failure primarily depends on the stress in fiber direction. In addition, a forced longitudinal stretching of the fiber due to radial pressure is considered. Equation (10) shows Puck’s criterion for fiber failure [27] (p. 410).

In an optimized resin cast anchorage, fiber failure is the leading failure mechanism. Nevertheless, very high transverse pressures can lead to splitting of the tendon or too small transverse pressure can lead to peeling (delamination) and, consequently, shear failure of the tendon. Both failure mechanisms are due to interfiber failure. Assuming a unidirectional material, interfiber failure occurs according to Puck always on a plane parallel to the fiber direction. Therefore, the tension in the fiber direction does not influence the interfiber failure. Equations (11) and (12) show the criteria for interfiber failure [27] (p. 421). It must be noted here that the stresses $\tau_{nt}$, $\tau_{n1}$ and $\sigma_{n}$ depend on the angle θ of the fracture plane and can be derived by a rotation matrix (13). According to Puck, the fracture plane is most likely where the exertion, i.e., $f_{E,Zfb}\left(\theta \right)$, is maximized. Therefore, one has to look for the highest value of $f_{E,Zfb}\left(\theta \right)$ and find the fracture plane’s orientation. Depending on the angle of the fracture plane, a distinction can be made between peeling and splitting of the tendon. If the fracture plane is tangential to the surface of the tendon, peeling can be assumed. If it is perpendicular, splitting will be activated. The individual stress states can be read from the output database file of the FE model. The Puck failure criterion was then evaluated with Matlab. The parameters used to calculate Puck’s failure criterion are summarized in Table A1 in Appendix A. Young’s modulus in fiber and transverse direction, reduced tensile strength and compressive strength perpendicular to the fibers were introduced in Section 2. The remaining values were estimated or come from the literature [27] (p. 428).

(10) $$f_{e,Fb}=\left|\frac{1}{R_{\|}^{\pm }}\left[\underset{\mathrm{Stress}\;\mathrm{in}\;\mathrm{fiber}\;\mathrm{direction}}{\underbrace{\sigma_{1}}}-\underset{\mathrm{Forced}\;\mathrm{longitudinal}\;\mathrm{stretching}}{\underbrace{\left(v_{⊥\|}-v_{f⊥\|}\frac{E_{\|}}{E_{f\|}}m_{\sigma f}\right)\left(\sigma_{2}+\sigma_{3}\right)}}\right]\right|$$

For $\sigma_{n}\geq 0$

(11) $$f_{e,Zfb}\left(\theta \right)=\sqrt{{\left[\left(\frac{1}{R_{⊥}^{+}}-\frac{p_{⊥\Psi }^{+}}{R_{⊥\Psi }^{A}}\right)\sigma_{n}\right]}^{2}+{\left(\frac{\tau_{nt}}{R_{⊥⊥}^{A}}\right)}^{2}+{\left(\frac{\tau_{n1}}{R_{⊥||}}\right)}^{2}}+\frac{p_{⊥\Psi }^{+}}{R_{⊥\Psi }^{A}}\sigma_{n}$$

For $\sigma_{n}<0$

(12) $$f_{e,Zfb}\left(\theta \right)=\sqrt{{\left(\frac{p_{⊥\Psi }^{-}}{R_{⊥\Psi }^{A}}\sigma_{n}\right)}^{2}+{\left(\frac{\tau_{nt}}{R_{⊥⊥}^{A}}\right)}^{2}+{\left(\frac{\tau_{n1}}{R_{⊥||}}\right)}^{2}}+\frac{p_{⊥\Psi }^{-}}{R_{⊥\Psi }^{A}}\sigma_{n}$$

(13) $$\left[\begin{array}{c} \sigma_{n}\\ \tau_{nt}\\ \tau_{n1} \end{array}\right]=\left[\begin{array}{ccccc} \cos{\left(\theta \right)}^{2} & \sin{\left(\theta \right)}^{2} & 2\cos(\theta )\sin(\theta ) & 0 & 0\\ -\cos(\theta )\sin(\theta ) & \cos(\theta )\sin(\theta ) & \left(\cos{\left(\theta \right)}^{2}-\sin{\left(\theta \right)}^{2}\right) & 0 & 0\\ 0 & 0 & 0 & \sin(\theta ) & cos(\theta ) \end{array}\right]\times \left[\begin{array}{c} \sigma_{2}\\ \sigma_{3}\\ \tau_{23}\\ \tau_{13}\\ \tau_{12} \end{array}\right]$$

**Figure 17:**
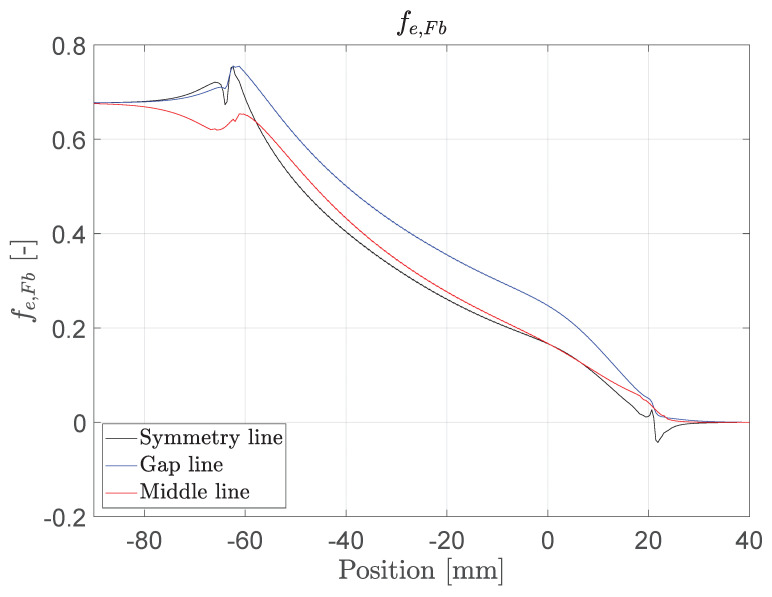
Fiber fracture exertion at σ=1856 MPa along the tendon.

The fiber fracture exertion at a tensile stress of σ=1856 MPa is shown in Figure 17. The stress peak at the front of the anchor is 12%. The exertion on the middle line is smaller, which is thought to be due to a shear lag. The results also show that the exertion is radius-dependent. The maximal exertion is smaller than 1, which indicates that other failure mechanisms are relevant. The focus lies here on the sand coating, which acts under high radial pressure as a “knife”.

**Figure 18:**
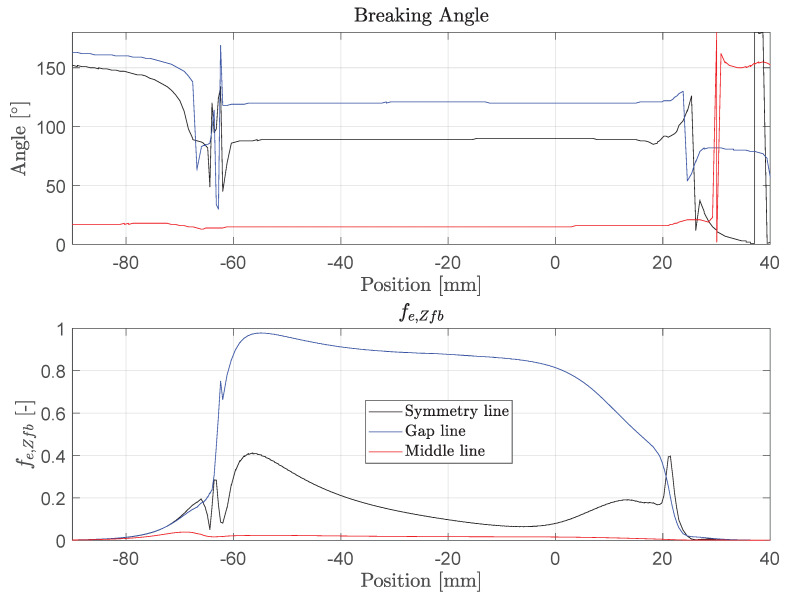
Interfiber fracture exertion and breaking angle at σ=1856 MPa along the tendon.

Interfiber failure exertion is shown in Figure 18. Apparently, no interfiber failure is expected at the middle line. The exertion at the gap line is close to 1. The breaking angle gives information about the failure mode. It must be noted here that the evaluation only makes sense in the area where $f_{e,Zfb}$ approaches the value 1. In this model, the definition of the fracture plane is as follows: an angle of 0° and 180° indicate peeling of the tendon, while an angle of 90° indicates splitting. On the gap line, we tend to have a mixed failure of splitting and peeling. On the symmetry line, splitting would be the leading failure mode, which is due to the high radial pressure.

No interfiber failure was reported in the experiment. High exertion on the gap line is thought to be due to the geometry of the wedge and the material model. The wedge central bore has a diameter of 7.5 mm, but the tendon has an outer diameter of 9 mm. This leads locally to high stresses. In reality, the wedge material shows non-linear deformations in this region, reducing the stress concentration. In addition, sand can be sheared off locally, which further reduces the stresses. Therefore, fiber failure due to local damage by the sand coating is the most probable failure cause, even if $f_{e,Fb}=0.75$ at maximum (see Figure 17). On the other hand, a combined fiber failure and mixed matrix tensile/shear failure at the loaded end of the anchor cannot be excluded.

## 6. Conclusions and Outlook

A wedge anchorage for the clamping of 7.5 mm tendons was successfully developed.

A finite element model was implemented in order to analyze the stress state in the tendon. The contact between the wedge and the sand-coated tendon was modeled by a pressure-overclosure condition and was determined based on circular samples with uncoated and coated CFRP lamellae. Puck’s failure criterion was used to predict fiber and interfiber failure. Tensile fiber failure at the loaded end of the anchor due to local damage by the sand coating was identified as the most probable failure cause.

A novel approach of producing tendons with integrated optical fiber was presented. It allows a high flexibility in the production of tendons and gives excellent measurement results. Samples with the developed wedge anchor were tested. They showed a stress peak on the front side of the anchorage of 17% and failed at an average stress of 1856 MPa, which is 68% of the assumed tensile strength. The measured strain distribution correlates with the finite element model.

In the future, we plan to further develop the FE model by implementing a non-linear viscoplastic material model of the wedge. Moreover, based on a refined reliable model, an extended parameter study and optimization could further improve the performance of anchors for sand-coated FRP tendons used in pretensioned precast concrete elements.

## Figures and Tables

**Table 1:** Tendon material properties.

Property	CFRP Tendon 7.5 mm
Effective diameter of tendon	7.5 mm ± 0.2 mm
Coated diameter	9.0 mm ± 0.2 mm
Carbon fiber	UTS50 F24 24K 1600tex by Toho Tenax (Japan)
Tensile strength fiber $\sigma_{f_{ult}}$	5000 MPa
Young’s modulus fiber $E_{f}$	245 GPa
Resin system	Epoxy Resin XB 3515/Aradur^®^ 1571 by Huntsman (Basel CH)
Young’s modulus resin $E_{m}$	2.85 GPa
Poisson’s ratio $v_{m}$	0.36

**Table 2:** Measured fiber volume content.

Sample	Fiber Volume Content $\psi_{f}$
Sample 1	61.34%
Sample 2	61.50%
Sample 3	61.26%
Sample 4	60.09%
Sample 5	60.52%
**Average**	60.94%
**Std. deviation**	0.61%

**Table 3:** Measured Young’s modulus.

Sample	Young’s Modulus $E_{11}$
Sample 1	155.3 GPa
Sample 2	147.6 GPa
Sample 3	144.8 GPa
**Average**	149.2 GPa
**Std. deviation**	5.4 GPa

**Table 4:** Tensile strength with resin cast anchorage.

Sample	Tensile Strength	Corresponding Tensile Stress
Sample 1	116.58 kN	2639 MPa
Sample 2	107.92 kN	2443 MPa
Sample 3	117.19 kN	2653 MPa
Sample 4	110.76 kN	2507 MPa
Sample 5	110.78 kN	2508 MPa
**Average**	112.65 kN	2550 MPa
**Std. deviation**	4.05 kN	91.6 MPa

**Table 5:** FE Model Parameters (* measured values).

	Barrel	Wedge	CFRP Tendon	Sand Layer
Orientation	Isotropic	Isotropic	Orthotropic	Orthotropic
$E_{1}$ [MPa]	210,000	4200	149,200 *	1
$E_{2}$ [MPa]	-	-	7407 *	1
$E_{3}$ [MPa]	-	-	7407 *	4866
$G_{12}$ [MPa]	-	-	3400	1000
$G_{13}$ [MPa]	-	-	3400	1000
$G_{23}$ [MPa]	-	-	2700	1000
$v_{12}$ [-]	0.3	0.35	0.26	0
$v_{13}$ [-]	-	-	0.26	0
$v_{23}$ [-]	-	-	0.39	0

**Table 6:** Description of the interactions in the model.

Interacting Parts	Interaction Type	Tangential Behavior	Normal Behavior
Barrel ↔ Wedge	master–slave	penalty = 0.2	hard contact
Wedge ↔ Sand layer	master–slave	penalty = 10	pressure-overclosure (see Figure 7c)
Sand layer ↔ Tendon	tie		

**Table 7:** Tensile strength with wedge anchorage.

Sample	Tensile Strength	Corresponding Tensile Stress
Sample 1	80.35 kN	1819 MPa
Sample 2	82.53 kN	1868 MPa
Sample 3	83.16 kN	1882 MPa
**Average**	82.01 kN	1856 MPa
**Std. deviation**	1.47 kN	33.4 MPa

## Data Availability

Part of the data can be found in the MSc thesis of Gian-Luca Züst submitted 2022 to the Laboratory of Composite Materials and Adaptive Structures of the Swiss Federal Institute of Technology in Zurich.

## Abbreviations

The following abbreviations are used in this manuscript:

CFRP	Carbon-Fiber-Reinforced Polymer
DFOS	Distributed Fiber Optical Sensing
Empa	Swiss Federal Laboratories for Materials Science and Technology
ETH	Eidgenössische Technische Hochschule (Swiss Federal Institute of Technology)
FE	Finite Element
FEM	Finite Element Method
GF	Glass fiber
PA	Polyamide
$R_{p0.2}$	Yield strength

## Appendix A. Implementing Puck’s Failure Criterion

**Table A1:** Typical values for implementing Puck’s failure criterion (* measured values).

Fiber failure index [-]	$f_{e,Fb}$	Greater than 1 means failure
Interfiber failure index [-]	$f_{e,Zfb}$	
Normal stress on the fracture plane (MPa)	$\sigma_{n}$	Calculated using the rotation matrix (13)
Transverse–transverse shear stress on the fracture plane (MPa)	$\tau_{nt}$	
Transverse–longitudinal shear stress on the fracture plane (MPa)	$\tau_{n1}$	
Axial stress (MPa)	$\sigma_{1}$	Resulting nodal stresses from the FE model
Tangential stress (MPa)	$\sigma_{2}$	
Radial stress (MPa)	$\sigma_{3}$	
Shear stresses (MPa)	$\tau_{12}$, $\tau_{13}$, $\tau_{23}$	
Tensile Strength in Fiber Direction (MPa)	$R_{\left|\right|}^{+}$	2742 *
Compressive Strength in Fiber Direction (MPa)	$R_{\left|\right|}^{-}$	1900
Tensile Strength of an UD Laminate Perpendicular to the Fibers (MPa)	$R_{⊥}^{+}$	57
Compressive Strength of an UD Laminate Perpendicular to the Fibers (MPa)	$R_{⊥}^{-}$	164 *
In-plane Shear Strength of an UD Laminate (MPa)	$R_{⊥}^{-}$	86
Inclination Parameter [-]	$p_{⊥\|}^{+}$	0.35
Inclination Parameter [-]	$p_{⊥\|}^{-}$	0.3
Inclination Parameter [-]	$p_{⊥⊥}^{+}$	0.275
Inclination Parameter [-]	$p_{⊥⊥}^{-}$	0.275
Poisson’s Ratio [-]	$v_{⊥\|}$	0.013
Poisson’s Ratio of the Fiber [-]	$v_{f⊥\|}$	0.1
Young’s Modulus in Fiber Direction (MPa)	$E_{\|}$	149,200 *
Young’s Modulus of the Fiber (MPa)	$E_{f\|}$	245,000
Magnification Factor [-]	$m_{\sigma f}$	1.1

Note: $R_{⊥⊥}^{A}$ is defined by $R_{⊥}^{-}$ and $p_{⊥\|}^{-}$ via Equation (A1) [27] (p. 426, Equation (17.49)).

(A1)
$$R_{⊥⊥}^{A}=\frac{R_{⊥}^{-}}{2(1+p_{⊥\|}^{-})}$$
